# The ovarian reserve as target of insulin/IGF and ROS in metabolic disorder-dependent ovarian dysfunctions

**DOI:** 10.1530/RAF-21-0038

**Published:** 2021-08-17

**Authors:** Maria Dri, Francesca Gioia Klinger, Massimo De Felici

**Affiliations:** 1 Department of Biomedicine and Prevention, University of Rome Tor Vergata, Rome, Italy

**Keywords:** metabolic disorders, female infertility, ovarian reserve, ROS, insulin/IGF

## Abstract

It is known for a long time that metabolic disorders can cause ovarian dysfunctions and affect a woman’s fertility either by direct targeting follicular cells and/or the oocytes or by indirect interference with the pituitary-hypothalamic axis, resulting in dysfunctional oogenesis. Such disorders may also influence the efficiency of the embryo implantation and the quality of the embryo with permanent effects on the fertility and health of the offspring. Thanks to the expanding knowledge on the molecular mechanisms governing oogenesis and folliculogenesis in mammals, we are beginning to understand how such disorders can negatively affect this process and consequently fertility in women. In the present review, we point out and discuss how the disturbance of insulin/IGF-dependent signalling and increased reactive oxygen species (ROS) level in the ovary typically associated to metabolic disorders such as type II diabetes and obesity can dysregulate the dynamics of the ovarian reserve and/or impair the survival and competence of the oocytes.

**Lay summary:**

In women, a progressive decline and depletion of the primary ovary reserve, which represents the reserve of immature eggs, are a challenging condition in the field of reproductive medicine. This decline, occurring physiological with age, is the main determinant of the age at the onset of menopause. Concomitant with the reduction in their number, the quality of the eggs also decreases with age. Metabolic disorders such as diabetes and obesity can cause ovarian dysfunctions and affect a woman’s fertility mainly by direct targeting the egg stockpile or by indirect interference with the production of reproductive hormones. Here, we report up-to-date data and discuss results about how disturbance of insulin-dependent signalling and increased oxidative stress in the ovary, usually associated to metabolic disorders, can dysregulate the dynamics of the primary ovary reserve and/or impair the survival and quality of the eggs.

## Introduction

Metabolic syndrome and infertility are two disorders with a high prevalence in the general population. This syndrome comprises distinct metabolic risk factors including obesity, hyperglycaemia, hypertriglyceridemia, hypertension, and low high-density lipoprotein cholesterol levels, that can increase the prevalence of type II diabetes and cardiovascular disease. The syndrome is also associated with proinflammatory state, prothrombotic state, non-alcoholic fatty liver disease, cholesterol gallstone disease, and, relevant for the present review, reproductive disorders. Metabolic syndrome has been reported in the reproductive literature to fall under a group of endocrine disturbances, including hypertension, obesity, dyslipidaemia, and insulin resistance. Actually, literature findings have demonstrated that conditions of negative energy balance and metabolic stress, including increased ROS, occurring in diabetes, acute inflammation, and chronic dietary restriction can affect fertility (Silvestris *et al.* 2019).

It is known for a long time that metabolic disorders can cause ovarian dysfunctions and affect a woman’s fertility either by direct targeting follicular cells and/or the oocytes or by indirect interference with the pituitary-hypothalamic axis, resulting in dysfunctional oogenesis. Such disorders may also influence the efficiency of the embryo implantation and the quality of the embryo with permanent effects on the fertility and health of the offspring. Thanks to the expanding knowledge on the molecular mechanisms governing oogenesis in mammals, we are beginning to understand how such disorders can negatively affect this process and consequently fertility in women.

In mammalian females, oocytes are formed before birth and are surrounded by somatic cells (pregranulosa cells) to form structures known as primordial follicles (PMFs). Oocytes entered meiosis and arrested at the dictyate stage of prophase I within the PMFs form the stockpile of female germ cells, termed ovarian reserve, will be utilised throughout reproductive life. Such reserve is progressively reduced with age leading to reproductive senescence. Follicles are gradually lost from the pool either through death (or atresia) or by activation of the growth pathway. Therefore, the rates of atresia and activation determine the size of the pool and the female fertility period. When the reserve reaches a certain critical threshold, women progress through the menopausal transition (onset of first menstrual irregularity, or skipped menses) until the ovarian reserve is completely depleted and, therefore, undergoes menopause.

In the present review, on the basis of the recent progresses in understanding the molecular mechanisms that regulate folliculogenesis in mammals, we highlight and discuss how disturbance of insulin/IGF-dependent signalling and increased ROS level in the ovary typically associated to metabolic disorders such as type II diabetes and obesity can dysregulate the dynamics of the ovary reserve and/or impair the survival and competence of oocytes.

## PI3K/Akt/mTORC1 pathways

Once PMFs of the ovarian reserve are recruited into the growing pool, the flattened pregranulosa cells differentiate to form a single layer of cuboidal cells surrounding the oocyte. In parallel, the oocyte increases in size and undergoes remarkable growth and maturation whilst still being maintained in meiotic arrest. These processes are referred to as PMF activation.

Each PMF has three possible developmental fates: (i) to remain quiescent (i.e. to survive in dormancy for various lengths of time throughout the reproductive period); (ii) to be activated into the growing follicle pool, that is either followed by atresia at a later stage of follicular development or by ovulation; (iii) to undergo death directly from the dormant state.

About the regulatory mechanisms of PMF activation, it can be basically recapitulated as the following three features: (a) external environment: the follicular microenvironment contains a variety of autocrine and paracrine growth factors that activate/inhibit specific signalling pathways in pregranulosa cells and/or oocytes and trigger or inhibit PMF activation. Among these factors, insulin/IGF and ROS associated to metabolic disorders such as type II diabetes and obesity can be included. (b) The communication between pregranulosa cells and oocytes: various hormones and growth factors produced by pregranulosa cells affect the development of oocytes. Conversely, the quality of the oocytes inversely influences the quality and quantity of pregranulosa cells, thus determining the activation, development, and apoptosis of follicles. (c) Physical and mechanical factors: the mechanical stimuli of the ovarian tissue, such as cell adhesion and the gradient of the ovarian tissue density, contributes to PMF through regulating signalling pathways including PI3K–AKT (phosphatidylinositol 3-Kinase- protein kinase B) and Hippo pathways.

To preserve the length of a woman’s reproductive life, it is essential that the majority of her PMFs are maintained in a quiescent state to provide a reserve for reproductive success. The molecular mechanisms underlying this lengthy quiescence and prolonged survival of PMFs have begun to reveal. These processes likely require a pregranulosa cell-oocyte regulatory loop in which bi-directional communication is essential.

Studies on mutant mouse models have demonstrated that the quiescence of PMFs is maintained by many molecules acting on pregranulosa and/or oocyte themselves.

It is likely that the transcription factor FOXL2 (forkhead box protein L2) regulates the transcription of key inhibitors in pregranulosa cells that via gap junctions or through paracrine factors suppress the initiation of oocyte growth. At the same time, AMH (anti-Müllerian hormone) produced by granulosa cells of secondary to early antral follicle acts on PMFs affecting the expression of a set of genes necessary for the transition from pre- to granulosa cells (Nilsson *et al.* 2011). Under physiological conditions, the activation of PMFs appears to initiate when mTORC1 (the mechanistic target of rapamycin 1 complex) is turned on in the pregranulosa cells (Zhang *et al.* 2014).

mTORC1 and its cognate mTORC2 are multiprotein complexes composed by the catalytic mTOR kinase and distinct protein partners (Saxton & Sabatini 2017). mTORC1 pathway integrates inputs from growth factors, stress, energy status, oxygen, and amino acids to control crucial cell processes such as protein and lipid synthesis and autophagy. TSC1/TSC2 (tuberous sclerosis complexes 1 and 2) is a key upstream regulator of mTORC1. Phosphorylation of this complex and mTORC1 itself by the effector kinases PI3K and Ras pathways lead to inactivation of the former and activation of the latter. mTORC1 controls protein synthesis through phosphorylation of eukaryotic translation initiation factor 4E (4E-BP1) and S6 kinase 1 (S6K1). When compared with mTORC1, though less understood than mTORC1, mTORC2 has been shown to respond to growth factors and to modulate cell metabolism and survival thanks to its capability to activate Akt, a serine/threonine-specific protein kinase that plays a key role in multiple cellular processes.

Zhang and colleagues found that when mTORC1 signalling in pregranulosa cells was suppressed, no or low levels of KITL (Kit ligand), also known as SCF (stem cell factor), were expressed and this was insufficient to activate the KIT receptor present on the oocyte membrane. In this situation, PI3K/PDK1 signalling within the oocyte is low and this maintains dormancy. Elevated mTORC1 signalling in the pregranulosa cells led to enhanced production of KITL that binds to KIT and subsequently activated the intraoocyte PI3K/PDK1/Akt signalling driving oocyte growth (Zhang *et al.* 2014).

In rat granulosa cells, KITL expression is increased by FSH and LH (Ismail *et al.* 1997), but other factors can be also involved. For example, an inhibitory effect of mouse oocyte on the KITL mRNA transcription in cumulus cells likely mediated by GDF9 (growth differentiation factor 9) has been reported (Joyce *et al.* 2000), although another oocyte-secreted factor BMP15 (bone morphogenetic factor 15) has been shown to stimulate cumulus KITL mRNA levels in rodents (Otsuka & Shimasaki 2002, Thomas *et al.* 2005, Miyoshi *et al.* 2012). Interestingly, IGF-1 was reported to induce KITL expression in zebrafish follicular cells (Yao *et al.* 2014).

Certainly, the oocyte status exerts a central role in the control of the PMF fate and PTEN (phosphatase and tensin homolog), a critical antagonist of the PI3K signalling pathway, and TSC1/TSC2 together with the transcription factor FOXO3a (forkhead box 3a), the CDK (cyclin-dependent kinase) inhibitor p27kip1 (p27), cooperate in maintaining oocyte quiescent. Notably, ablation of *Pten*, *Tsc1*-*Tsc2*, *Foxo3a* or *p27* genes in mouse oocytes resulted in rapid global activation of PMFs. In this regard, a critical role of the PI3K/Akt/mTORC1 signalling pathway has been revealed. In the quiescent oocytes, this pathway is negatively regulated by PTEN and TSC1/2. When PTEN or TSC1/2 is eliminated, Akt and/or mTORC1 are activated, and consequently, phosphorylation and cytoplasmic sequestration of FOXO3a and p27 by Akt as well, activation of S6K1 by mTORC1 occur driving the oocyte growth (Jagarlamudi *et al.* 2009, Adhikari *et al.* 2010, Tanaka *et al.* 2012). It is to mention that in humans, the incubation of ovarian tissue in the presence of PTEN inhibitor or PI3K activator generates massive PMF growth initiation (Li *et al.* 2010, Kawamura *et al.* 2013, Lerer-Serfaty *et al.* 2013, McLaughlin *et al.* 2014, Novella-Maestre *et al.* 2015). On the other hand, high PI3K/Akt activity is linked to a decline in the number of PMFs and ovarian ageing (Reddy *et al.* 2008, 2009).

Furthermore, a basal Akt activity likely provided by mTORC2 seems to be necessary for oocytes for its and PMF survival. In fact, when PI3K/PDK1/Akt signalling is abolished in oocytes, all PMFs were prematurely lost directly from their quiescent state (Reddy *et al.* 2010).

As reported above, under physiological conditions, the activation of PMFs appears to initiate in the pregranulosa cells following activation of mTORC1. Factors or conditions leading to this initial activation in the selected PMFs remain elusive. Considering that primordial to early antral follicle development is largely gonadotrophin-independent, other hormones and local growth factors are believed to exert such functions. At the same time, these factors can impact the PI3K/Akt/mTORC1 signalling of the dormant oocyte and facilitate or inhibit such process.

For example, the notion that the growth hormone (GH) influences numerous processes associated with ovarian function including PMF activation is relatively old (Silva *et al.* 2009). Actually, results obtained in the mice lacking the GH receptor (GJR) or the GH binding protein showed that the hormone may play a role in the recruitment of PMFs into the growing pool (Slot *et al.* 2006). In primates, including humans, the mRNA for GHR has been found in pregranulosa cells, whereas positive immunostaining for GHR was detected in both oocytes and pregranulosa cells of PMFs (Abir *et al.* 2008). It is unclear, however, whether the effect of the absence of GH signalling is directly or indirectly a result of reduced IGF-I signalling. This appears subject to further modulation through the local elaboration of low-molecular-weight binding proteins (IGFBPs), the role and regulation of which by GH are receiving increasing attention. For instance, IGFBP-1 being down-regulated by insulin has an important role in the pathophysiology of polycystic ovary syndrome (PCOS) by increasing the free, biological active IGF-I and augmenting the androgen generation in the theca layer (Blumenfeld 1994).

IGF-I is a potent mitogen and regulator of cellular survival and apoptosis for a wide variety of cells including granulosa cells, predominantly by activating the PI3K/Akt intracellular signalling. Clear evidence for the central role of IGF-I in reproductive physiology has been gained from gene knockout technology. In the mouse, targeted null mutation of the *Igf1* gene encoding IGF-I results in infertility secondary to failure to ovulate even after administration of gonadotropins (Baker *et al.* 1996).

It is now well established that the ovary is a site of IGF-I gene expression and reception. Expression of IGF-I and its receptors has been shown primarily in theca and granulosa cells; however, IGF-1 gene expression and IGF-1 type 1 receptor were found also in oocytes within PMFs or other classes of follicles of various mammals, including humans (Silva *et al.* 2009, Poljicanin *et al.* 2015). IGF is known to stimulate the proliferation of granulosa and theca cells and enhances the ability of gonadotropins to stimulate steroidogenesis in these cell populations. Furthermore, IGF has a direct antiapoptotic effect and is selectively expressed in healthy follicles compared with atretic follicles (Louhio *et al.* 2000).

Relevant for the present review, the addition of IGF-1 to the medium of ovarian tissue culture has been reported to promote PMF activation in caprine and ovine (Martins *et al.* 2010, Luz 2013, Costa 2014) and human follicles (Louhio *et al.* 2000). Moreover, Bezerra *et al.* (2018), reported that IGF-1 promoted activation of ovine PMFs* in vitro*, stimulating at the same time granulosa cell proliferation and reducing DNA fragmentation in such cells through the PI3K/Akt pathway. In this regard, it is worthy to mention that in a model of mouse gestational diabetes, the PMF formation in the ovaries of a newborn in which the levels of serum glucose and insulin were higher than in control were significantly increased via upregulating the PI3K/AKT signalling pathway (Xu *et al.* 2019). In line with this, in another paper, it was reported that insulin promoted PMF assembly and activation process via the insulin receptor/PI3K/Akt signalling pathway in E 16.5 foetal mouse ovaries cultured* in vitro* but it repressed the phosphorylation of Akt and follicular assembly and activation in the 3 days post-partum cultured ovaries (Zhang *et al.* 2010).

It is likely that since mTORC1 can sense and be activated by a variety of microenvironment changes several elements including stress factors, oxygen and energy conditions are involved. Supporting such a possibility, the Hippo pathway, ROS and local glucose concentration have been found to play a role in PMF activation. Relevant for the present review is that insulin/IGF system can have a modulatory and sometimes critical role in all these factors.

## The Hippo pathway

Hippo signalling is an evolutionarily conserved pathway that controls organ size. The major functions of this signalling are to restrict tissue growth in adults and modulate cell proliferation, differentiation, and migration in developing organs (Misra & Irvine 2018). Core to the Hippo pathway is a kinase cascade in which MST1/2 (mammalian sterile20-like 1/2, ortholog of Drosophila Hippo) kinases and SAV1 (Salvador family WW domain-containing protein 1) form a complex to phosphorylate and activate LATS1/2 (large tumor suppressor 1 and 2). In turn, LATS1/2 kinases phosphorylate and inhibit the transcription co-activators YAP1 (Yes-associated protein1) and TAZ (transcriptional coactivator with PDZ-binding motif), two major downstream effectors of the Hippo pathway. The Hippo pathway can be regulated by a variety of factors, such as cell–cell and cell-extracellular matrix contacts, cell polarity, and actin cytoskeleton, as well as a wide range of other signals, including the cell energy status, mechanical cues, hormones and growth factor signals.

In the ovaries, YAP1 is localised in the cytoplasm of PMF pregranulosa cells and secondary follicles (Hu *et al.* 2019, Lv *et al.* 2019). First studies from the Hsueh’s laboratory demonstrated that temporary activation of YAP1 by physical fragmentation of ovaries or use of chemical treatments could promote ovarian follicle growth (Kawamura *et al.* 2013, Cheng *et al.* 2015). In a whole-body *Lats1*-knockout mouse model, LATS1 was found to play a role in maintaining the ovary reserve (Sun *et al.* 2015). Under physiological conditions, follicular development in the ovary is accompanied by a reduction in MST1 and LATS2 and elevation in YAP1 expression, accompanied by a decrease in MST1 phosphorylation, indicating its inactivation (Hu *et al.* 2019). YAP1 is spatiotemporally expressed in the granulosa cells and plays a critical role in granulosa cell proliferation, differentiation and survival (Lv *et al.* 2019). Moreover, YAP1 knockdown resulted in a significant elevation in the number of PMFs accompanied by a decrease in the number of primary follicles. Conversely, a reduced number of PMFs and elevation in secondary follicles were obtained upon YAP1 overexpression (Xiang *et al.* 2015, Hu *et al.* 2019). Other findings revealed that the Hippo-YAP1 regulates PMF activation, through AKT signalling in mice (Hu *et al.* 2019). The mechanical stimuli of the ovarian tissue, such as cell adhesion and the gradient of the ovarian tissue density, contribute to primordial follicle activation (Woodruff & Shea 2011, Yin *et al.* 2016) through regulating signalling pathways including the Hippo pathway and the PI3K–AKT pathway (Cheng *et al.* 2015, Hsueh *et al.* 2015). Interestingly, YAP has been shown to be responsible for the transcription of the microRNA mir.29, which in turn inhibits the translation of PTEN. This down-regulation of PTEN by YAP1 leads to increased PI3K signalling and subsequent increased activation of mTORC1 and mTORC2 (Tumaneng *et al.* 2012).

Some findings demonstrate that IGF-1R may act as a critical upstream modulator of Hippo-YAP signalling and that IGF-1 promoted YAP expression in some cell types (Zhou *et al.* 2020). Thus suggesting that the insulin/IGF system could influence PMF activation through the Hippo pathway.

## Glucose metabolism

Glucose uptake and glycolysis by granulosa cells are crucial for providing energy for the maturing oocytes. While growing oocytes preferentially metabolise pyruvate over glucose, the somatic compartment of ovarian follicles is more glycolytic. Furthermore, the intricate metabolic relationship between each oocyte and its somatic surroundings is critical for oocyte growth and developmental competence. Quiescent and early stage oocytes seem able to use glycolysis and the Krebs cycle for energy metabolism rather than oxidative phosphorylation to avoid the consequences of reactive oxygen species generated from such process and maintain PMF integrity (Cinco *et al.* 2016). Interestingly, IGF1 has been reported to act in an autocrine manner on granulosa cells, and in a paracrine manner on oocytes to increase the expression of the glucose transporter GLUT1 (Zhou *et al.* 2000).

Besides trophic action, the correct intake of glucose by granulosa cells seems to be crucial for PMF activation. In fact, very recently, Xu and colleagues (Xu *et al.* 2020) reported that glucose concentration affected the activation of mouse PMF both* in vitro* and* in vivo* through the AMPK/mTOR signalling pathway. They found that when the concentration of glucose in the culture medium or in blood was below threshold levels, the expression of AMPK (AMP-activated protein kinase) in the ovary was high while the mTOR pathway was inactive and PMF activation was prevented. Interestingly, in a model of diabetic mice, oocytes showed deregulated AMPK activity.

It is well known that one of the major causes of type II diabetes and obesity is an abnormality in glucose metabolism and glucose uptake in a variety of cell types. Therefore, it is possible to postulate that defects in glucose uptake in pregranulosa cells can result in low cytoplasmic glucose and reduced PMF activation impairing the ovulatory performance. In this regard, it is now clear that in women with PCOS, usually associated with insulin resistance, as well as with defects in insulin secretion, ovarian reserve is better preserved than in normo-ovulatory women of similar age.

## ROS

High levels of reactive oxygen species (ROS) are intricately linked to obesity and associated pathologies, notably insulin resistance and type II diabetes. Reactive oxygen species (ROS) include superoxide anion radicals (O^.^_2_^−^), hydroxyl radicals (OH^−^), hydrogen peroxide (H_2_O_2_), and other peroxides. ROS are formed through the leakage of electrons from the inner mitochondrial membrane during oxidative phosphorylation and ATP generation. In steroidogenic tissues such as the ovary, steroidogenic cytochrome P450 enzymes are also sources of ROS. While many studies in recent years have shown that H_2_O_2_ and other ROS act as important signalling molecules within cells, mitochondrial dysfunction can result in excessive ROS accumulation. At the cellular level, high levels of ROS cause various oxidative injuries, such as lipid peroxidation of cell membranes, enzyme inactivation, protein oxidation and DNA damage.

At the level of the ovary, ROS may have a regulatory role in folliculogenesis, ovarian steroidogenesis, luteolysis and oocyte maturation whereas excessive ROS induce inflammation and oxidative stress (Wang *et al.* 2017). Actually, there is a delicate balance between ROS and antioxidant enzymes in the ovarian tissues (Wang *et al.* 2017). In this regard, it has been reported that in women, high ROS levels in the follicular microenvironment reduce ovarian reserve and decrease ovarian functions (Pertynska-Marczewska & Diamanti-Kandarakis 2017).

About the ovarian reserve, excessive ROS could accelerate its depletion either by favouring PMF activation and inducing degeneration in pregranulosa cells and/or oocytes. In this regard, it is interesting to observe that Shimamoto *et al.* (2019) showed that hypoxia through hypoxia-inducible factors (HIFs) and FOXO3a contributed to maintain the dormant state of oocytes in cultured mouse ovaries (Shimamoto *et al.* 2019). Under such condition, HIFs and genes related to response to OS downstream of FOXO3a are likely to attenuate ROS production. Detoxification of ROS is particularly important in oocytes as they are the sole cell type to generate the next generation. The dormant state can be established under hypoxia, but at the same time, it must protect the quality of the oocytes against OS. About favouring PMF activation, high ROS might inactivate the Hippo pathway (see above) as it has been reported in some cell types (Mao *et al.* 2015).

On the other hand, the importance of ROS and OS in ovarian toxicity by diverse stimuli is well documented. There is strong evidence that ROS are involved in the initiation of apoptosis in antral follicles upon gonadotropin hormone withdrawal and exposure to chemicals including phthalates, polycyclic aromatic hydrocarbons (PAHs), methoxychlor, cyclophosphamide and ionising radiation (Luderer 2014). Although less attention has been focused on the roles of ROS in primordial and primary follicle atresia, several studies have shown protective effects of antioxidants and/or evidence of oxidative damage, suggesting that ROS may play a role in these follicle classes as well. Exposures to agents known to cause OS, such as gamma irradiation, chemotherapeutic drugs, heavy metal Cr or PAHs, induce rapid PMF loss; however, the mechanistic role of ROS has received limited attention (Luderer 2014).

One of the most dangerous effects of high ROS on cells is the damage to genomic and mitochondrial DNA. In fact, ROS not only destroy DNA bases to generate 7,8-dihydro-8-oxo-2-deoxyguanosine (8-oxodG) but also produce spontaneous DNA double-strand breaks (DSBs). Although, cells with unrepairable DSBs are normally eliminated by apoptosis, DSBs can lead to several types of DNA mutation, including nonsense, missense, frameshift, deletion, and translocations in surviving cells. These are particularly risky effects in oocytes fated to transmit both genomic and mitochondrial DNA to progeny.

In this regard, oocytes within PMF express in the nucleus, high amount of inactive TAp63α, a transcription factor belonging to the p53 family. This factor and under certain circumstances, p53 itself become rapidly activated in the oocytes following IR or exposure to chemotherapy agents causing DSBs (Rinaldi *et al.* 2020). Experiments with mice have demonstrated a direct connection between the expression of TAp63α and the induction of apoptosis following DSBs. The mechanism of induction of apoptosis in oocytes by TAp63α has been described in several studies. The two BH3-only proteins, PUMA and NOXA, are direct transcriptional targets of p63. Their combined effect of inhibiting the pro-survival family member BCL-2 and activation of pro-apoptotic family member BAX results in oocyte death (Spears *et al.* 2019, Martinez-Marchal *et al.* 2020).

Although IR and chemotherapeutic that cause TAp63 activation and apoptosis are able to increase ROS levels, as far as we know, a direct effect of ROS on TAp63 or p53 of the oocyte was not investigated. Similarly, if OS and inflammation, commonly occurring in obesity and diabetes, can induce damage in genomic and mitochondrial DNA and/or inhibit DNA repair mechanisms in oocytes within PMF remain to be addressed.

Furthermore, recent evidence indicate that ROS are able to induce epigenetics alteration (DNA methylation and histone modifications) but how this occurs are still unclear (Davalli *et al.* 2016). Certainly, considering the importance of epigenetics for oocyte maturation, such a possibility rises alarming perspectives and must be verified.

Finally, an increasing number of studies have focused on Sirtuins (SIRTs) a family of NAD^+^-dependent deacetylases as key metabolic sensors for body homoeostasis. These enzymes respond to metabolic challenges, inflammatory signals or hypoxic/oxidative stress. SIRTs have recently been linked to the mTOR signalling pathway in rat ovaries, capable of targeting the activation inhibitor FOXO3a in order to control PMF activation in response to environmental cues like nutrient status (Tatone *et al.* 2018).

## Conclusions

**Figure 1:**
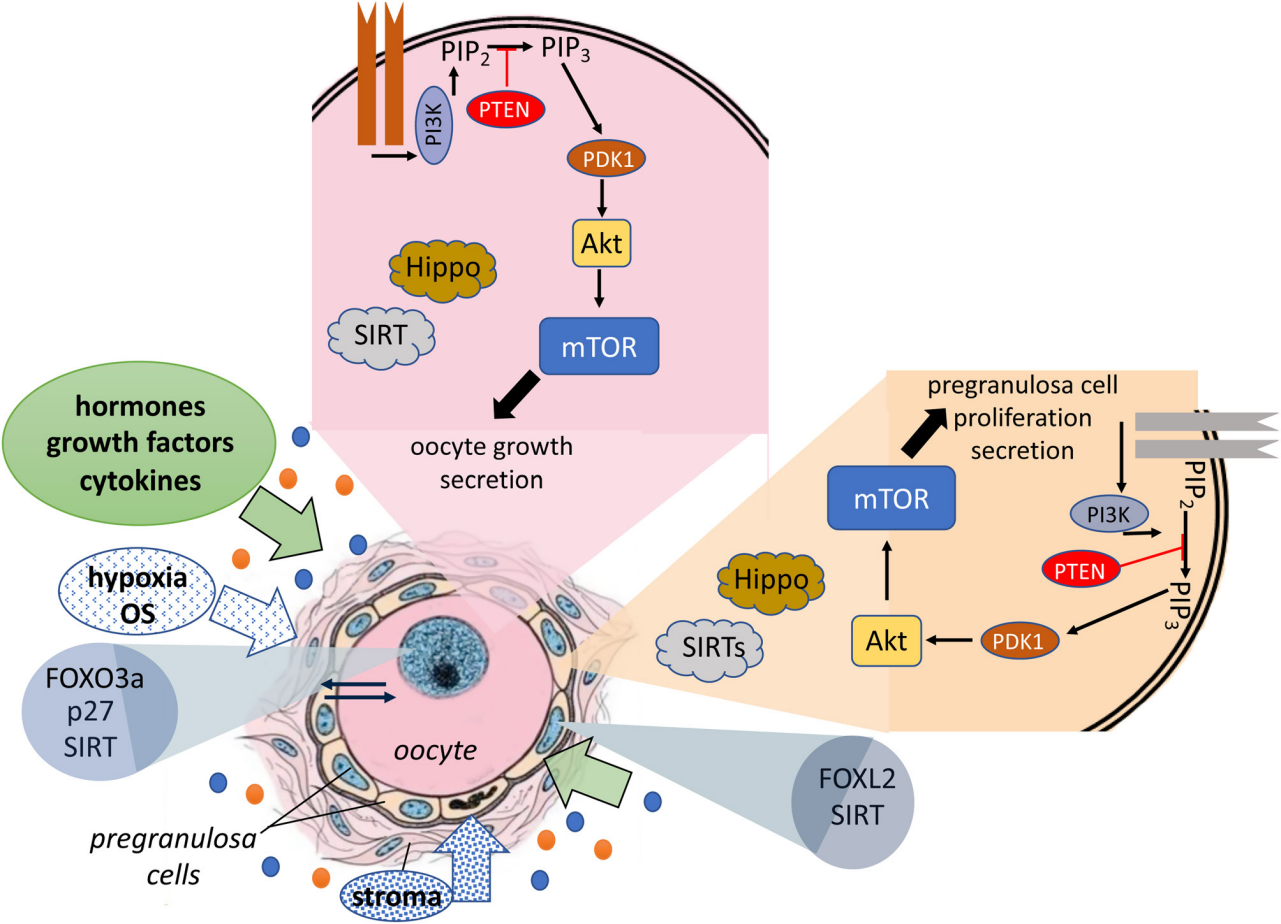
Schematic representation of the main players regulating the PF quiescence and activation. The current hypothesis of PF activation in mice follows that mTORC1 is activated in pregranulosa cells of PFs and then KL produced by activated granulosa cells then activates the oocyte via PI3K/Akt also converging on mTORC1 activation. Various factors including soluble molecules (hormones, cytokines, growth factors), stroma components, hypoxia and oxidative stress can positively or negatively contribute to PF quiescence/activation acting on pregranulosa cells or oocytes or both.

While the regulation of PMF activation is highly complex and finely orchestrated by the microenvironment (including local growth factor concentration, hypoxia, glucose availability, mechanical cues) (Fig. 1), it is important to consider that their aberrant activation contributes to the diminishment of the ovarian reserve over the life and leads to pathological conditions such as ovarian insufficiency/premature ovarian failure (POI/POF). Regardless of the mechanisms, when the reserve reaches a certain critical threshold, women progress through the menopausal transition (onset of first menstrual irregularity, or skipped menses) until the ovarian reserve is completely depleted and, therefore, undergoes menopause.

The balance among PMF survival/atresia and activation ultimately determines the reproductive performance of females in mammals. Among potential novel intraovarian regulators, growth factors, cytokines, and neuropeptides have been the subject of increasingly intense investigation. Most of these agents are not expected to act in the traditional endocrine fashion because of their local intraovarian generation. Speculation supports the notion that intraovarian regulators may engage in refined but important *in situ* modulation and coordination of functions of the different ovarian cell types, mainly granulosa cells and oocytes.

Hyperinsulinaemia and insulin resistance are recognised features of polycystic ovary syndrome (PCOS) and there is evidence that augmentation, in granulosa cells, of the action of luteinising hormone (LH) by insulin is implicated in the mechanism of arrested follicle development that is characteristic of anovulation in PCOS. Recent data suggest that insulin resistance in PCOS selectively affects the metabolic action of insulin on pyruvate production by granulosa cells, a mechanism likely to be mediated by the activity of the PI3K/Akt pathway. Obesity is a leading factor of insulin and IGF resistance along with chronic inflammation. Obesity leads to a low-grade inflammatory response to the adipose tissue so setting the activation of the immune cells. These are likely responsible for the overstimulation of cytokines leading to insulin resistance and IGF-1 resistance. In a more complex scenario, obesity causes changes in the gut microbiota resulting in increased epithelial permeability, lipopolysaccharide leak and activation of Toll-like receptor signalling in the gut and increased production of short-chain fatty acids as well; together, these lead to inflammation and oxidative stress and ovarian dysfunction (Robker *et al.* 2011, Snider & Wood 2019). Moreover, it is now clear that OS influences the entire reproductive span of a women’s life. There is some understanding of how ROS affect a variety of ovarian functions such as PMF activation, follicle and oocyte maturation, ovarian steroidogenesis, ovulation and luteolysis. Future studies could involve altering the expression of protective antioxidant defence mechanisms specifically in small follicles or oocytes using available transgenic mice. Further research measuring the effects of toxicant exposure on ROS generation and the modulation of toxicant effects by antioxidant supplementation or depletion in isolated cultured follicles and ovaries would also help in characterising small follicle sensitivity. In addition, future studies could investigate the effect of insulin-sensitisers in mouse models in order to better understand the insulin/IGF-dependent signalling in the ovary (Ahmadian *et al.* 2013). In this regard, Sirtuins, a family of NAD^+^-dependent deacetylases that catalyse post-translational modifications of proteins are emerging as an important regulator of insulin metabolism/sensitivity and oxidative stress in several organs including the ovary (Revollo & Li 2013, Tatone *et al.* 2018).

## Declaration of interest

Francesca Gioia Klinger is an Associate Editor of Reproduction and Fertility. Francesca Gioia Klinger was not involved in the review or editorial process for this paper, on which she is listed as an author. The other authors declare no conflict of interest.

## Funding

This work did not receive any specific grant from any funding agency in the public, commercial or not-for-profit sector.

## Author contribution statement

All authors contributed to literature analysis and drafting of the article. M D F and F G K contributed to the study design, as well as critical reading and editing.
